# Progress in Surgery of the Stomach

**Published:** 1903-01-31

**Authors:** 


### PROGRESS IN SURGERY OF THE STOMACH.

(*Concluded from page 286.*)

**Bilocular or Hour-glass Stomach.**—Cumston[4] says this condition may be congenital or acquired. The former origin has been explained in various ways; thus, Cruveilhier assumes an analogy with the stomach of ruminants, and considers it an example of atavism; Guillemot ascribes it to an abnormal arterial supply of the organ, and Carrington discusses the possibility of some cases being produced by an abscess during fœtal life. The most important etiological factor of the acquired condition is the contraction consequent upon the healing of a gastric ulcer. Other causes are the corrosion of the gastric mucous membrane by the ingestion of acids or alkalies, syphilitic lesions of the stomach and also carcinomatous affections. Rosmussen directs attention to the fact that the cicatrix often follows the line of the pressure sulcus of the liver upon the stomach, and he considers that pressure-necrosis along this line may explain some cases of bilocular stomach. Most frequently the stomach is divided into two fairly equal cavities, but in several instances the cardiac pouch has been found much the larger. In reported cases the lumen of the connecting channel varies from the size of a lead-pencil to that of admitting four fingers. A splashing sound is common, but merely indicates that the organ is dilated. If, however, care be taken to pass the stomach-tube previously, and syphon off the contents as far as possible, and, after this, a splashing sound is obtained, there is a strong probability in favour of bilocular stomach. Several procedures have been advocated, but the most generally applicable is gastro-gastrostomy (gastro-anastomosis), failing which gastro-enterostomy between the cardiac pouch and jejunum or duodenum, as circumstances permit, is advisable. Non-operative gastrolysis (breaking down of adhesions) consists in frequently inflating the stomach so as to stretch or tear down adhesions to surrounding viscera. Büdinger[5] reports three cases of operation for "hour-glass stomach" in which gastro-enterostomy gastric resection, and gastro-plasty were respectively performed, the last proving fatal. In two of the cases abdominal palpation, by alternately pressing with one hand on either side of the abdomen, gave a distinct feeling as though fluid were being forced through a small aperture. Elder[6] reports a successful case of gastro-plasty for this condition, and Gilford[7] has had three similarly successful cases and quotes Robson, who has collected 13 cases of which 11 recovered.

**Gastrectomy.**—Greig[8] says that, according to Linossier, there were over twenty successful cases of total gastrectomy reported in 1901. The pancreas assumes the saponification of fat after gastrectomy. Bœkel[9] reports a case of cancer of the stomach in which the tumour removed weighed 275 grams, and comprised the entire stomach with the exception of the left cul-de-sac and the first part of the duodenum. The man was 36 years old and had suffered for five years but had never had vomiting, hæmatemesis, or melæna. Syme[10] also reports a successful case of gastrectomy for carcinoma of the pylorus and lesser curvature of the stomach.

**Gastric Ulcer.**—Howitt[11] assumes that the essential cause of gastric ulceration is the presence of a micro-organism. He states that by the intelligent use of the $x$-rays when the stomach contains subnitrate of bismuth in suspension we may generally distinguish between pyloric stenosis and hour-glass contraction of the stomach. He says success in operation depends largely on early recognition and promptness of action, but the size and situation of the perforation, the nature of the infectious material present, the duration of the time after the last meal, and the perfection of the technique of operation are also important factors. There are no reliable symptoms that are indicative of impending perforation, but fortunately in over 90 per cent. of the cases there is a previous history of dyspeptic troubles. When it occurs, the most reliable of the early symptoms in the acute form, are sudden onset of excrutiating pain in the epigastric region, shock generally of a severe character, rigidity of abdominal walls, marked suppression of abdominal respiratory movements, arrest of peristalsis, and shortly, all those of acute septic peritonitis. As a rule, the pulse and temperature do not indicate the gravity of the trouble until too late for the surgeon to act with reasonable success. Excision of the ulcer is recommended, and thorough flushing with normal saline solution of all parts, including the lesser sac of the peritoneum. When drainage is necessary, it should be effected through stab-wounds made for the purpose. In desperate cases a pint of milk is injected into the jejunum, and when constipation is a factor a saline cathartic is similarly injected into the ascending colon. Cases of operation for gastric ulcer are reported by Thomas,[12] Barker,[13] Moullin,[14] Heaton,[15] and others.

[4] Med. Chron., April 1902, p. 50. [5] Ibid., p. 51. [6] Annals of Surg., May 1902, p. 508. [7] Brit. Med. Jour., Nov. 8, 1902, p. 1527. [8] Scottish Med. and Surg. Jour., June 1902, p. 549. [9] Med. Rec., Sept 27, 1902, p. 510. [10] Lancet, Sept. 13, 1902, p. 735. [11] Quart. Med. Jour., May 1902, p. 275. [12] Lancet, Aug. 16, 1902, p. 441. [13] Lancet, Aug. 23, 1902, p. 501. [14] Lancet, July 5, 1902, p. 17. [15] Brit. Med. Jour., July 12, 1902, p. 96.

